# Clinical course as a core feature of bipolar disorder

**DOI:** 10.1192/j.eurpsy.2026.10373

**Published:** 2026-07-31

**Authors:** M. Alda

**Affiliations:** 1 National Institute of Mental Health, Klecany, Czech Republic; 2 Psychiatry, Dalhousie University, Halifax, Canada

## Abstract

**Background:**

Bipolar disorder (BD) has been variably conceptualized as a recurrent/episodic or a chronic condition. This lack of consensus may reflect the heterogeneity of the illness, potentially shaped by external factors such as treatment effects, comorbid conditions, or early-life trauma.

**Methods:**

We present longitudinal data from 799 patients with BD meeting DSM-IV criteria. Intake assessments were conducted using the SADS-L or SCID interviews and subsequently reviewed in a blinded manner by a panel of expert clinicians. Information on clinical course, symptom profiles, and comorbidities was obtained through interviews, OPCRIT ratings, and medical records. Family history was assessed using direct interviews with relatives and the FH-RDC. After bivariate analyses, polychotomous logistic regression was performed with type of clinical course as the dependent variable, along with factor analysis of clinical profiles using the principal component method. A second cohort of 128 patients was used to assess the validity of the findings.

**Results:**

The sample included 313 men and 486 women, with a mean age at last assessment of 45.1 ± 15.0 years and a mean illness duration of 20.7 ± 13.5 years. Of these, 549 patients had BD type I and 250 had BD type II. Clinical course was classified as episodic with full symptomatic and functional recovery between episodes (n = 311), episodic with residual symptoms and/or incomplete functional recovery (n = 237), or chronic (n = 251). The three groups differed significantly (all ps < 0.001) across multiple variables, including BD subtype, age at onset, predominant polarity, long-term lithium response, family history of BD, and comorbid conditions. Across comparisons, the episodic group with residual symptoms showed intermediate characteristics between the fully episodic and chronic groups. In logistic regression analyses, an episodic course was associated with BD type I diagnosis, manic predominant polarity, lower rates of comorbid generalized anxiety disorder (GAD) and obsessive-compulsive disorder (OCD), and a positive family history of BD. Factor analysis identified six factors: (F1) diagnosis, polarity, and psychotic symptoms; (F2) social anxiety and GAD; (F3) clinical course and family history; (F4) panic disorder and OCD; (F5) sex and age at onset; and (F6) substance abuse. These findings were largely replicated in the second cohort.

**Conclusions:**

These results support the view that patients with an episodic clinical course represent a distinct subtype of BD characterized by stronger genetic liability and better long-term response to lithium treatment. Future studies should investigate these differences at the genomic level.

**Disclosure of Interest:**

None Declared

